# Validation of KIDMED 2.0 PL—Mediterranean Diet Quality Index for Polish Children and Adolescents

**DOI:** 10.3390/nu17162636

**Published:** 2025-08-14

**Authors:** Julia Bober, Ewelina Gaszyńska

**Affiliations:** 1 International Doctoral School, Medical University of Lodz, 90-647 Lodz, Poland; 2 Department of Nutrition and Epidemiology, Medical University of Lodz, 90-752 Lodz, Poland

**Keywords:** mediterranean diet, BMI, KIDMED, validation, children, adolescents, eating behaviours

## Abstract

Background: The Mediterranean diet is widely recognised for its health benefits and remains a key reference point in shaping dietary guidelines across populations. Despite its growing international relevance, there is a lack of validated tools assessing Mediterranean diet adherence among children and adolescents in Central and Eastern Europe. Methods: The present study aimed to adapt and validate the KIDMED 2.0 questionnaire for use in Polish children and adolescents aged 10 to 18 years (KIDMED 2.0 PL). The adaptation process involved forward–backward translation, expert consultations, and pilot testing to ensure linguistic and cultural relevance. A total of 102 participants completed the questionnaire twice over a two-week interval, and anthropometric data were collected. Results: The KIDMED 2.0 PL demonstrated high test–retest reliability (Spearman’s ρ = 0.876; *p* < 0.001) and strong criterion validity, with a significant negative correlation between KIDMED scores and BMI centile (ρ = −0.854; *p* < 0.001). Children with normal weight showed the highest adherence to the Mediterranean diet, while scores were significantly lower in overweight and obese participants. Item-level analysis indicated that fruit and vegetable consumption was relatively frequent, whereas intake of legumes, whole grains, and extra virgin olive oil remained low. Conclusions: The KIDMED 2.0 PL is a valid and reliable tool for evaluating diet quality and Mediterranean dietary adherence in the Polish pediatric population.

## 1. Introduction

The Mediterranean diet represents a nutritional model associated with numerous health benefits and is widely recognised for its emphasis on the consumption of fresh, minimally processed foods [1,2]. Its core components include a high intake of vegetables, fruits, legumes, whole grains, and nuts, with moderate consumption of fish and dairy products, particularly fermented ones such as yoghurt and cheese [3,4]. Red meat intake is limited, while virgin olive oil is the primary source of dietary fat [5]. In addition, this dietary pattern discourages the intake of ultra-processed foods, sugary snacks, and sweetened beverages [6]. Recent studies suggest that diet quality among children and adolescents across Europe has declined, with increasing intake of processed foods and sugar-sweetened beverages [7]. In Poland, over 30% of school-aged children are classified as overweight or obese, raising concerns about long-term health outcomes, including 35.6% of eight-year-olds measured in the World Health Organisation (WHO) Childhood Obesity Surveillance Initiative (COSI) Round 6 [8].

A characteristic feature of the Mediterranean dietary approach is the prioritisation of seasonal and local ingredients, culinary diversity, and culturally rooted eating behaviours [9]. However, in recent years, the adoption of traditional Mediterranean dietary habits has declined significantly, especially among younger populations [10]. Factors contributing to this shift include rapid urbanisation, changes in family structures, and the widespread availability of highly processed convenience foods [11,12]. These changes have resulted in a nutritional transition marked by excessive consumption of sugar-rich products, refined cereals, and fast food, which displace healthier, traditional alternatives [13].

In light of these developments, growing attention has been directed toward reversing these unfavourable trends, particularly due to the escalating prevalence of childhood overweight and obesity observed in Mediterranean and non-Mediterranean countries [14]. Recent reports indicate a high prevalence of excess body weight among children in several Central and Eastern European regions, including Poland [8]. In this context, the Mediterranean diet has been highlighted not only as a preventive model but also as a benchmark for defining healthy eating habits [15]. Therefore, in order to evaluate diet quality and adherence to this nutritional pattern in children and adolescents, brief and validated dietary screening tools are being implemented in public health research [16].

Despite the widespread use of the original KIDMED index in assessing adherence to the Mediterranean diet among youth populations, the updated version, KIDMED 2.0, has not yet been adapted, culturally validated, or tested for psychometric properties within a Polish context [17,18]. Given the importance of ensuring both linguistic accuracy and cultural relevance in dietary assessment tools, the present study was undertaken to (i) adapt the KIDMED 2.0 questionnaire to the Polish language and dietary context (KIDMED 2.0 PL) and (ii) evaluate its reliability and validity in a sample of Polish children and adolescents aged 10 to 18 years.

The validation process included a forward–backward translation, expert consultation, and a pilot study to assess comprehension. The test–retest reliability was measured over a two-week interval, and criterion validity was assessed based on anthropometric measurements and Body Mass Index (BMI) centile classification. This study aims to provide a stable, reliable, and culturally appropriate instrument for assessing adherence to the Mediterranean diet among Polish youth.

## 2. Materials and Methods

### 2.1. Study Design and Participants

**Figure 1:**
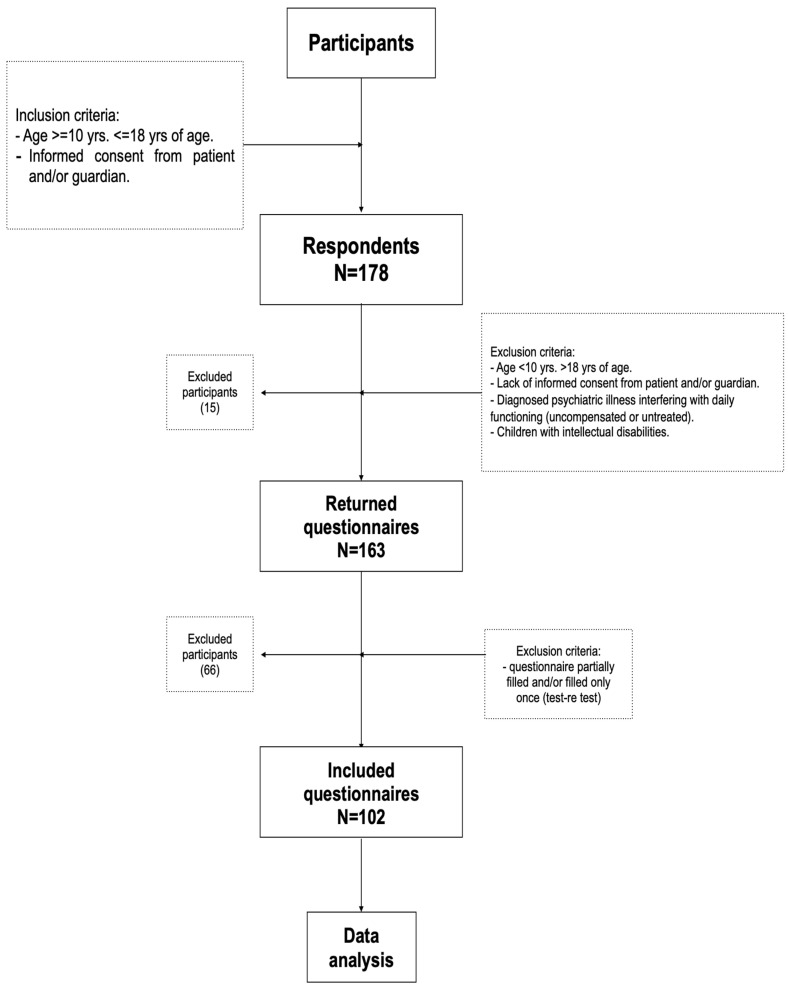
Flowchart. Study design and data collection (N-number of participants).

This cross-sectional validation study was conducted among Polish children and adolescents aged 10–18 years. The sample size was determined based on previous validation studies of dietary screening tools in youth populations, including previous versions of the original KIDMED [16,17,18]. Previous studies typically used samples ranging from 80 to 150 participants, which we considered sufficient for the psychometric aims of this research. Participants (N = 102) were recruited from public and private schools and local communities in various regions of Poland (Mazowieckie, Łódzkie, and Małopolskie voivodeships). These regions cover both urban and semi-urban areas with varying levels of access to health education and nutritional support. Although detailed individual-level socioeconomic data were not collected, the inclusion of both public and private schools aimed to capture a range of educational and socioeconomic contexts. The online nature of data collection may have favoured families with higher digital access, which is addressed in the study limitations. Eligibility criteria included (i) age between 10 and 18 years; (ii) Polish nationality; and (iii) informed consent by both the participant and their parent or legal guardian. Exclusion criteria involved any chronic illness or medical condition that could influence dietary intake or nutritional status (Figure 1).

Anthropometric data were collected for all participants, including body weight and height, and used to calculate BMI. These data were self-reported by participants (or reported by their parents/guardians for younger children). BMI centiles were determined based on the World Health Organisation (WHO) growth reference standards for age and gender. Participants were classified into underweight, normal weight, overweight, and obese groups.

### 2.2. Questionnaire Adaptation

In adapting the KIDMED 2.0 questionnaire to the Polish context, several modifications were introduced to ensure linguistic clarity, cultural relevance, and dietary specificity (Table 1). The original English version was translated using a forward–backward method, followed by expert review. During this process, emphasis was placed on reflecting typical Polish dietary habits, food availability, and terminology.

Several items were adjusted or expanded. The item assessing the use of olive oil was modified to include a reference to commonly used oils in Poland, sunflower and rapeseed oil, providing clearer contrast for respondents. Breakfast-related items were refined to evaluate the quality of breakfast consumption, rather than simply its presence or absence.

Protein-related questions were consolidated to better align with the Polish meal structure, which often combines lean meats and eggs in daily meals. Food examples were adapted throughout the questionnaire (e.g., inclusion of oats, buckwheat, and rye-based products) to ensure cultural familiarity.

These adaptations were made in consultation with dietitians, pediatricians, and public health experts to preserve the conceptual framework of the original tool while enhancing its applicability in the Central and Eastern Europe context.

The KIDMED 2.0 PL questionnaire consists of 16 items: 12 items reflect positive adherence to the Mediterranean dietary pattern and are scored +1 point for a “Yes” response, while 4 items (questions: 6, 12, 14, 16) reflect negative dietary behaviours and are scored −1 point. The total score ranges from −4 to +12, with higher scores indicating greater dietary adherence. Based on established cut-off points, participants were categorised into three adherence levels: low (≤3 points), moderate (4–7 points), and high (≥8 points) (Supplementary Materials).

### 2.3. Pilot Testing

A pilot study was conducted with a convenience sample of 10 Polish children and their parents to evaluate the comprehension, relevance, and acceptability of the translated tool. Feedback was analysed and used to make minor modifications to question wording and formatting to ensure clarity and ease of administration.

### 2.4. Statistical Analysis

#### 2.4.1. Test–Retest Reliability

The collected data were subjected to statistical analysis. To assess temporal stability, participants completed the KIDMED 2.0 PL questionnaire twice, with a two-week interval between administrations. The test–retest reliability was evaluated using Spearman’s rank correlation coefficient, with *p* < 0.05 considered statistically significant.

#### 2.4.2. Criterion Validity

Criterion-related validity was assessed by examining the association between KIDMED 2.0 PL scores and BMI centile values. Spearman’s rho was calculated to determine the strength and direction of correlations. Additionally, mean questionnaire scores were compared across BMI-defined weight status categories using the Kruskal–Wallis test.

### 2.5. Ethical Approval and Consent to Participate

This study was conducted in accordance with the Declaration of Helsinki. Ethical approval was obtained from the Bioethics Committee of the Medical University of Łódź (RNN/04/24/KE). Written informed consent was obtained from all participants and their legal guardians prior to data collection.

## 3. Results

### 3.1. Sample Characteristics

The final sample comprised 102 children and adolescents aged 10 to 18 years (M = 13.8; SD = 2.6) (Table 2). Participants were stratified into four weight-status groups based on BMI centile according to WHO growth standards: underweight (n = 12), normal weight (n = 30), overweight (n = 30), and obese (n = 30). Girls accounted for 53.9% of the total sample. No statistically significant age differences were observed between weight categories.

### 3.2. Adherence to the Mediterranean Diet by Weight Status

KIDMED 2.0 PL scores differed substantially across BMI categories. Children with normal weight achieved the highest mean scores (M = 8.6; SD = 1.7), followed by underweight participants (M = 7.0; SD = 1.5) (Table 3). In contrast, lower scores were observed in the overweight (M = 3.7; SD = 0.8) and obese groups (M = 1.7; SD = 1.4). A Kruskal–Wallis test confirmed statistically significant differences in KIDMED 2.0 PL scores across weight-status groups (H = 38.72, *p* < 0.001). Post hoc comparisons using Dunn’s test indicated that both overweight and obese groups scored significantly lower than normal-weight participants (*p* < 0.001).

### 3.3. Distribution of KIDMED 2.0 PL Scores

Analysis of score distribution revealed that over half of the participants (52.9%) demonstrated low adherence to the Mediterranean diet (KIDMED 2.0 PL score < 3) (Table 4). Approximately 28.5% achieved moderate adherence (scores 4–7), while only 18.6% met the criteria for high adherence (score ≥ 8). This trend was consistent across both genders.

### 3.4. Reliability and Validity

The KIDMED 2.0 PL showed high stability (Table 5). Test–retest reliability assessed over a two-week interval yielded Spearman’s correlation coefficient of ρ = 0.876 (*p* < 0.001), indicating strong reproducibility of responses. The criterion validity was supported by a significant inverse correlation between KIDMED 2.0 PL scores and BMI centile (ρ = −0.854; *p* < 0.001), suggesting that a lower adherence to the Mediterranean diet was associated with higher BMI centile values.

### 3.5. Selected Dietary Behaviours

The most commonly reported positive behaviours included daily fruit consumption (72%) and regular vegetable intake (65%) (Table 6). Only 12% of participants consumed legumes at least three times per week, and 19% reported the use of extra virgin olive oil or virgin rapeseed oil at home. Regarding negative dietary indicators, 74% of children consumed sweets or industrially processed snacks several times per week, and 61% drank store-bought juices or sweetened beverages at least once per week.

## 4. Discussion

To our knowledge, this is the first study to validate the updated KIDMED 2.0 questionnaire in the Polish population. The results showed that the Polish version (KIDMED 2.0 PL) is both reliable and valid. The tool showed high test–retest stability and strong inverse correlations with BMI centile, meaning it accurately reflects diet quality and its link to weight status. These findings are in line with previous Mediterranean-based studies, which have shown the KIDMED 2.0 to be a solid tool for assessing children’s diet [16,17,18,19,20,21].

Although the Mediterranean diet is rooted in southern Europe, its core principles, such as increased consumption of fruits, vegetables, legumes, nuts, and healthy fats, are relevant everywhere, especially in the context of rising childhood obesity [21,22,23,24,25,26]. In our sample, the level of adherence was generally low. Only about one in five participants met the criteria for high adherence to the Mediterranean diet, and more than half had low scores. Similar results have been reported in other countries, suggesting that the diets of young people are moving away from traditional healthy patterns [24,25,26,27,28].

Our results confirmed that lower KIDMED scores were linked with higher BMI centiles. Children with obesity had the lowest scores, while normal-weight participants had the highest. This is consistent with research showing that children who eat more processed and high-energy foods are more likely to gain excess weight [29,30,31,32,33,34,35].

Looking at individual items, we observed that while fruit and vegetable intake was common, other important components of the Mediterranean diet, like legumes and extra virgin olive oil, were rarely consumed. This might be due to cultural preferences, food availability, or even cost [36,37,38,39,40,41]. At the same time, many children reported frequently eating sweets and drinking sugary beverages. These habits, which are typical of highly processed Western-style diets, have been linked to weight gain and other health issues in young people [42,43,44,45,46,47].

These patterns suggest that adopting the Mediterranean diet in Poland may be challenging due to structural and behavioural barriers. In Central and Eastern Europe, traditional ways of eating are being replaced by more processed and convenience foods. Compared to countries like Spain, where foods such as olive oil and legumes are more common, the Polish diet may not naturally follow Mediterranean-style eating [17,19,21,22,34]. Still, similar studies from other non-Mediterranean countries like Turkey, Romania, Lithuania, and Norway have also found low adherence among young people, showing that this is a wider trend in many parts of Europe [48,49,50,51,52].

These findings point to the importance of early dietary assessment and education. Tools like KIDMED 2.0 PL can help identify children with poor diet quality and support targeted actions, particularly in schools [53,54,55]. Since diet and emotional wellbeing are closely connected during childhood and adolescence, future interventions should also address emotional eating and stress. This makes the KIDMED 2.0 PL a useful resource not just for researchers, but also for educators, dietitians, and public health professionals working with children in Poland. Nonetheless, certain limitations should be acknowledged. The sample, while diverse, was limited and may not reflect all Polish children and adolescents. While recruitment included schools from regions with diverse population density and schooling models, the absence of detailed sociodemographic variables such as parental income, education, or rural/urban classification limits deeper analysis. In addition, the online nature of data collection may have excluded families with limited digital access or engagement, introducing potential socioeconomic selection bias. Dietary data as well as height and weight were self-reported by participants/or parents, which may introduce bias. Additionally, other lifestyle factors, such as physical activity and sleep, were not assessed.

## 5. Conclusions

This study confirmed that the adapted KIDMED 2.0 PL questionnaire is a reliable and valid tool for assessing adherence to the Mediterranean diet among Polish children and adolescents. Low adherence to the Mediterranean dietary pattern was strongly associated with overweight and obesity, particularly among children with higher BMI centile values. In conclusion, this study highlights the potential of the Mediterranean diet as a benchmark for healthy eating in youth, even in non-Mediterranean regions. The validated KIDMED 2.0 PL can serve as a screening tool in schools to monitor dietary quality and in public health programmes to identify unhealthy eating patterns and support nutrition education initiatives. Further research is needed to monitor long-term changes in dietary adherence and assess how these behaviours influence weight, metabolic health, and psychosocial outcomes throughout childhood and adolescence.

## Figures and Tables

**Table 1:** Comparison of the original KIDMED 2.0 and the Polish-adapted KIDMED 2.0 PL.

Item No.	KIDMED 2.0 (Original, English)	KIDMED 2.0 PL (Adapted, English Translation)	Scoring	Adaptation Type
1	Consumes two or more servings of fruit per day.	My child consumes two or more servings of fruit per day.	+1	Direct translation
2	Consumes at least one serving of vegetables per day.	My child consumes at least one serving of raw and/or cooked vegetables per day.	+1	Cultural contextualisation
3	Eats legumes, lean meat, fish, or eggs at lunch and dinner.	My child consumes one portion of legumes, lean meat, fish, and/or eggs at lunch and another one at dinner.	+1	Content clarification
4	Diet is mostly plant-based (fruits, vegetables, legumes, nuts, whole grains).	More than half of my child’s diet consists of plant-based products (fruits, vegetables, legumes, nuts, potatoes, whole grains).	+1	Cultural contextualisation
5	When consuming meat, fish, or eggs, they are fresh and minimally processed.	When my child consumes lean meat, eggs, and/or fish, these are usually fresh and minimally processed.	+1	Direct translation
6	Eats pre-prepared meals or fast foods (e.g., pizza, burgers) one or more times per week.	My child consumes pre-prepared meals or fast food (e.g., pizza, hamburgers) one or more times per week.	−1	Cultural contextualisation
7	Eats three or more servings of legumes per week.	My child eats at least three servings of legumes (chickpeas, beans, soy, lentils, peas) per week.	+1	Content clarification
8	Food is usually baked, grilled, or boiled instead of deep-fried.	At home, meals are usually prepared by baking, grilling (with little oil), or boiling (not deep-frying).	+1	Cultural contextualisation
9	Chooses whole grains when eating cereals, pasta, rice, etc.	When my child eats cereal-based foods (pasta, rice, groats), these are usually whole-grain products.	+1	Cultural contextualisation
10	Eats nuts ≥3 times/week without added salt.	My child eats a portion (min. 15 g) of natural or roasted unsalted nuts at least three times per week.	+1	Content clarification
11	Uses extra virgin olive oil at home.	At home, we use extra virgin olive oil (dark green) or virgin rapeseed oil (dark yellow) instead of sunflower or rapeseed oil (bright yellow).	+1	Cultural contextualisation
12	Drinks commercial juices, nectars, or soft drinks one or more times per week.	My child drinks store-bought beverages, juices, nectars, and/or smoothies one or more times per week.	−1	Content clarification
13	Dairy products are natural or minimally processed (e.g., milk, yoghurt, fresh cheese).	My child always chooses natural or minimally processed dairy products (milk, unsweetened yoghurt, fresh cheese).	+1	Direct translation
14	Eats pastries, cookies, or drinks processed beverages for breakfast.	For breakfast, my child eats pastries, cookies, and/or drinks juices, smoothies, or other processed products.	−1	Cultural contextualisation
15	Eats whole or minimally processed foods for breakfast (fruit, vegetables, eggs, whole-grain bread, etc.).	For breakfast, my child eats unprocessed or minimally processed foods (e.g., fruits, vegetables, oats, eggs, whole-grain bread).	+1	Content clarification
16	Eats sweets, cookies, or processed snacks several times per week.	My child consumes industrially processed sweets (candies, cookies, snacks, chocolate) and/or desserts (chips, cakes, jelly candies) more than once a week.	−1	Cultural contextualisation

**Table 2:** Sociodemographic characteristics of the study sample by weight status.

Variables	Underweight (n = 12)	Normal Weight (n = 30)	Overweight (n = 30)	Obese (n = 30)	Total (N = 102)
mean age (SD), years	15.0 (2.4)	13.5 (2.5)	13.6 (2.7)	13.5 (2.9)	13.8 (2.6)
girls, n (%)	6 (50.0%)	17 (56.7%)	16 (53.3%)	19 (63.3%)	55 (53.9%)
boys, n (%)	6 (50.0%)	13 (43.3%)	14 (46.7%)	11 (36.7%)	47 (46.1%)

**Table 3:** KIDMED 2.0 PL scores by weight status.

Weight Status	n	Mean KIDMED 2.0 PL Score	Standard Deviation (SD)	Statistical Comparison
Underweight	12	7.0	1.5	Underweight and normal weight scored significantly higher on the KIDMED 2.0 PL than overweight and obese.
Normal weight	30	8.6	1.7	
Overweight	30	3.7	0.8	
Obese	30	1.7	1.4	
Kruskal–Wallis H test				H = 38.72, *p* < 0.001

**Table 4:** Distribution of KIDMED 2.0 PL scores among participants (N = 102).

Adherence Level	Score Range	n	% of Total Sample
low adherence	≥3	54	52.9%
moderate adherence	4–7	29	28.5%
high adherence	≥8	19	18.6%
total	—	102	100.0%

**Table 5:** Test–retest reliability and criterion validity of the KIDMED 2.0 PL.

Measure	Statistic	Value	Interpretation
test–retest (Time 1)	Mean ± SD	5.24 ± 2.89	–
test–retest (Time 2)	Mean ± SD	5.31 ± 2.92	–
reliability	Spearman’s ρ	0.876 (*p* < 0.001)	High reproducibility over a 2-week interval
criterion validity	Spearman’s ρ (KIDMED vs. BMI centile)	−0.854 (*p* < 0.001)	Strong inverse correlation

**Table 6:** Frequencies of selected KIDMED 2.0 PL items reported by participants (N = 102).

Item No.	KIDMED 2.0 PL Item (English Translation)	Type	n (%) Responding “Yes”
1	My child consumes two or more servings of fruit per day.	Positive (+1)	74 (72.5%)
2	My child consumes at least one serving of raw and/or cooked vegetables per day.	Positive (+1)	66 (64.7%)
7	My child eats at least three servings of legumes per week.	Positive (+1)	12 (11.8%)
11	At home, we use extra virgin olive oil (dark green) or virgin rapeseed oil (dark yellow) instead of sunflower or rapeseed oil (yellow).	Positive (+1)	19 (18.6%)
16	My child consumes industrially processed sweets and/or desserts several times per week.	Negative (−1)	76 (74.5%)
12	My child drinks store-bought beverages, juices, nectars, and/or smoothies one or more times per week.	Negative (−1)	62 (60.8%)

## Data Availability

The data that support the findings of this study are available on request from the corresponding author, J.B.

## Supplementary Materials

The following supporting information can be downloaded at https://www.mdpi.com/article/10.3390/nu17162636/s1: Validated version of KIDMED 2.0 PL, Mediterranean Diet Quality Index for Polish Children and Adolescents.

## Abbreviations

The following abbreviations are used in this manuscript:

WHO	World Health Organisation
COSI	Childhood Obesity Surveillance Initiative
KIDMED	Mediterranean Diet Quality Index
KIDMED 2.0 PL	Mediterranean Diet Quality Index for Polish children and adolescents
BMI	Body Mass Index
