# Supplementary material for: Validation of KIDMED 2.0 PL—Mediterranean Diet Quality Index for Polish Children and Adolescents
Source: Nutrients. 2025 Aug 14;17(16):2636. doi: 10.3390/nu17162636 (PMC12389672; doi:10.3390/nu17162636)
Supplement: Supplementary file 1 [file nutrients-17-02636-s001.zip › nutrients-3816637-supplementary.pdf]

# KIDMED 2.0 PL$_{\text{KID}}$

KIDMED 2.0 PL służy do badania poziomu przestrzegania diety śródziemnomorskiej u dzieci i młodzieży. Narzędzie to składa się z 16 pytań opartych na ocenie nawyków żywieniowych, z których 12 odnosi się do pozytywnych nawyków, a 4 do negatywnych. Wskaźnik przestrzegania diety śródziemnomorskiej jest obliczany jako suma wszystkich odpowiedzi i może wynosić od -4 do 12.

**KWESTIONARIUSZ WYPEŁNIANY PRZEZ DZIECKO:**

1. Jem dwie lub więcej porcji owoców dziennie (0/+1).

2. Jem jedną lub więcej porcji surowych i/lub gotowanych warzyw dziennie (0/+1).

3. Jem codziennie jedną porcję roślin strączkowych, mięsa (kurczaka, indyka, królika lub innego chudego mięsa), ryb i/lub jajek na obiad i drugą porcję na kolację (0/+1).

4. Ponad połowa spożywanej przeze mnie żywności jest pochodzenia roślinnego (owoce, warzywa, rośliny strączkowe, orzechy, ziemniaki, produkty pełnoziarniste) (0/+1).

5. Kiedy jem chude mięso, jaja i/lub ryby, są one zazwyczaj świeże i minimalnie przetworzone (0/+1).

6. Jem wstępnie przygotowane posiłki lub produkty typu fast-food, takie jak pizza i hamburgery, jeden lub więcej razy w tygodniu (0/-1).

7. Jem trzy lub więcej porcji roślin strączkowych (ciecierzyca, fasola, soja, soczewica, groch) tygodniowo (0/+1).

8. W domu jedzenie jest zazwyczaj przygotowywane w piekarniku, grillowane (na patelni z niewielką ilością oleju) lub gotowane (bez smażenia w głębokim tłuszczu) (0/+1).

9. Kiedy jem produkty zbożowe (makaron, ryż, kasze), zazwyczaj są to produkty pełnoziarniste (0/+1).

10. Jem porcję naturalnych lub prażonych orzechów bez dodatku soli co najmniej 3 razy w tygodniu (0/+1).

11. W domu używamy oliwy z oliwek z pierwszego tłoczenia (ciemnozielonej, extra virgin) lub oleju rzepakowego tłoczonego na zimno, zamiast oleju słonecznikowego i rzepakowego (jasno-żółtego) (0/+1).

12. Piję dostępne w sklepach napoje, soki, nektary i/lub koktajle jeden lub więcej razy w tygodniu (0/-1).

13. Kiedy jem porcję produktów mlecznych, zawsze są to produkty naturalne (mleko, jogurt bez cukru, świeży ser) lub minimalnie przetworzone (0/+1).

14. Na śniadanie jem wyroby cukiernicze, ciastka i/lub pije soki, koktajle lub produkty przetworzone (0/-1).

15. Na śniadanie jem produkty nieprzetworzone lub minimalnie przetworzone (owoce, warzywa, orzechy, płatki owsiane, jajka lub pieczywo pełnoziarniste) (0/+1).

16. Jem przemysłowe wyroby cukiernicze (cukierki, ciastka, przekąski lub czekoladę) i/lub desery (chipsy, ciasta lub żelki) częściej niż raz w tygodniu (0/-1).

**Wynik wskaźnika KIDMED 2.0 PL**
Niski (≤3)
Średni (4-7)
Prawidłowy (≥8)

# KIDMED 2.0 $PL_{Parent}$

KIDMED 2.0 służy do badania poziomu przestrzegania diety śródziemnomorskiej u dzieci i młodzieży. Narzędzie to składa się z 16 pytań opartych na ocenie nawyków żywieniowych, z których 12 odnosi się do pozytywnych nawyków, a 4 do negatywnych. Wskaźnik przestrzegania diety śródziemnomorskiej jest obliczany jako suma wszystkich odpowiedzi i może wynosić od -4 do 12.

**KWESTIONARIUSZ WYPEŁNIANY PRZEZ RODZICA:**

1. Moje dziecko je dwie lub więcej porcji owoców dziennie (0/+1).

2. Moje dziecko je jedną lub więcej porcji surowych i/lub gotowanych warzyw dziennie (0/+1).

3. Moje dziecko je codziennie jedną porcję roślin strączkowych, mięsa (kurczaka, indyka, królika lub innego chudego mięsa), ryb i/lub jajek na obiad i drugą porcję na kolację (0/+1).

4. Ponad połowa spożywanej przez moje dziecko żywności jest pochodzenia roślinnego (owoce, warzywa, rośliny strączkowe, orzechy, ziemniaki, produkty pełnoziarniste) (0/+1).

5. Kiedy moje dziecko je chude mięso, jaja i/lub ryby, są one zazwyczaj świeże i minimalnie przetworzone (0/+1).

6. Moje dziecko je wstępnie przygotowane posiłki lub produkty typu fast-food, takie jak pizza i hamburgery, jeden lub więcej razy w tygodniu (0/-1).

7. Moje dziecko je trzy lub więcej porcji roślin strączkowych (ciecierzyca, fasola, soja, soczewica, groch) tygodniowo (0/+1).

8. W domu jedzenie jest zazwyczaj przygotowywane w piekarniku, grillowane (na patelni z niewielką ilością oleju) lub gotowane (bez smażenia w głębokim tłuszczu) (0/+1).

9. Kiedy moje dziecko je produkty zbożowe (makaron, ryż, kasze), zazwyczaj są to produkty pełnoziarniste (0/+1).

10. Moje dziecko je porcję (min.15g) naturalnych lub prażonych orzechów bez dodatku soli co najmniej 3 razy w tygodniu (0/+1).

11. W domu używamy oliwy z oliwek z pierwszego tłoczenia (ciemnozielonej, extra virgin) lub oleju rzepakowego tłoczonego na zimno, zamiast oleju słonecznikowego i rzepakowego (jasno-żółtego) (0/+1).

12. Moje dziecko pije dostępne w sklepach napoje, soki, nektary i/lub koktajle jeden lub więcej razy w tygodniu (0/-1).

13. Kiedy moje dziecko je porcję produktów mlecznych, zawsze są to produkty naturalne (mleko, jogurt bez cukru, świeży ser) lub minimalnie przetworzone (0/+1).

14. Na śniadanie moje dziecko je wyroby cukiernicze, ciastka i/lub pije soki, koktajle lub produkty przetworzone (0/-1).

15. Na śniadanie moje dziecko je produkty nieprzetworzone lub minimalnie przetworzone (owoce, warzywa, orzechy, płatki owsiane, jajka lub pieczywo pełnoziarniste) (0/+1).

16. Moje dziecko je przemysłowe wyroby cukiernicze (cukierki, ciastka, przekąski lub czekoladę) i/lub desery (chipsy, ciasta lub żelki) częściej niż raz w tygodniu (0/-1).

**Wynik wskaźnika KIDMED 2.0 PL**
Niski (≤3)
Średni (4-7)
Prawidłowy (≥8)
